# Inhibition effect of *Caragana sinica root* extracts on Osteoarthritis through MAPKs, NF-κB signaling pathway

**DOI:** 10.7150/ijms.52330

**Published:** 2021-01-01

**Authors:** Ga-Yul Min, Jong-Min Park, In-Hwan Joo, Dong-Hee Kim

**Affiliations:** Department of Pathology, College of Oriental Medicine, Daejeon University, Daejeon 34520, Republic of Korea.

**Keywords:** Osteoarthritis (OA), *Caragana sinica root* (CSR), monosodium iodoacetate (MIA), interleukin (IL)-1β, extracellular matrix (ECM), matrix metalloproteinases (MMPs)

## Abstract

Osteoarthritis (OA) is a common joint disease characterized by degradation and inflammation of cartilage extracellular matrix. We aimed to evaluate the protective effect of *Caragana sinica root* (CSR) on interleukin (IL)-1β-stimulated rat chondrocytes and a monosodium iodoacetate (MIA)-induced model of OA. *In vitro*, cell viability of CSR-treated chondrocytes was measured by MTT assay. The mRNA expression of Matrix metallopeptidases (MMPs), a disintegrin and metalloproteinase with thrombospondin motifs (ADAMTSs) and extracellular matrix (ECM) were analyzed by quantitative real-time PCR (qRT-PCR). Moreover, the protein expression of MAPK (phosphorylation of EKR, JNK, p38), inhibitory kappa B (IκBα) and nuclear factor-kappa B (NF-κB p65) was detected by western blot analysis. *In vivo*, the production of nitric oxide (NO) was detected by Griess reagent, while those of inflammatory mediators, MMPs and ECM were detected by ELISA. The degree of OA was evaluated by histopathological analyses, Osteoarthritis Research Society International (OARSI) score and micro-CT analysis. CSR significantly inhibited the expression of MMPs, ADAMTSs and the degradation of ECM in IL-1β-stimulated chondrocytes. Furthermore, CSR significantly suppressed IL-1β-stimulated of MAPKs, NF-κB signaling pathway. *In vivo*, CSR and Indomethacin inhibited the production of inflammatory mediators, MMPs and degradation of ECM in MIA-induced model of OA. In addition, CSR improved the severity of OA. Taken together, these results suggest CSR is a potential therapeutic active agent in the treatment of OA.

## Introduction

Osteoarthritis (OA) is the chronic degenerative disease among the aging population. The main feature of OA is joint pain, dysfunction, and subchondral osteophyte formation by degeneration of articular cartilage with degradation of the cartilage matrix^1^-^3^. Articular cartilage contains chondrocytes that are associated with the synthesis and degradation of Extracellular matrix (ECM) 4, 5. The main components of the ECM are Type II collagen, Aggrecan, and Proteoglycans 6. Recent studies have reported that production of pro-inflammatory mediators is involved in the development of OA 7. Interleukin (IL)-1β is a pro-inflammatory cytokine, which plays an important catabolic role in ECM degradation and the pathological development of OA 8. Elevated IL-1β levels stimulate transcription factor (NF-κB), a downstream signaling molecule 9-11. NF-κB activation contributes to increase pro-inflammatory cytokine release 12. Mitogen-activated protein kinase (MAPK) is involved in the regulation of NF-κB activity in cartilage destruction 13, 14. The MAPK/NF-κB signaling pathway is important in the pathogenesis of OA and increased release of catabolic enzymes such as Matrix metalloproteinases (MMPs), and a disintegrin and metalloproteinase with thrombospondin motifs (ADAMTSs) 15, 16. Increased MMPs downregulate the synthesis of ECM including Type II collagen, Aggrecan, Glycosaminoglycans (GAGs), and proteoglycans by inhibiting the anabolic activity of chondrocytes 17. Furthermore, IL-1β increases the expression of potential COX-2 and iNOS and increases the synthesis of NO and PGE2 18. Thus, inhibiting MMPs synthesis and blocking ECM destruction may protect cartilage degradation and be beneficial for OA treatment.

Currently, steroidal and Nonsteroidal anti-inflammatory drugs (NSAIDs) are commonly used as pharmacological drugs to alleviate OA symptoms, especially in patients that have undergone joint replacement surgery 19, 20. However, the long-term use of these drugs cannot complete prevent OA progression and cause serious side effects such as cardiovascular disease, gastrointestinal toxicity, and weakened renal function 21, 22. Nutraceuticals, glucosamine, and chondroitin sulfate have also been used as OA symptom relievers. Recent studies have reported that using it alone or in combination with glucosamine is not effective 23, 24. Therefore, to prevent OA progression and improve side effects, new drugs must be discovered. Herbal medicine with anti-inflammatory effects, low toxicity, and lower cost are attractive options for treating OA.

Caragana sinica root (CSR), the dried root of Caragana sinica (Buc'hoz) Rehder, is part of the Caragana genus belonging to the Fabaceae family 25. The dried roots of CSR have been used as an effective folk remedy in Korea for treatment of neuralgic, rheumatic, and arthritic conditions 26. In addition, CSR has been reported to have anti-apoptotic, anti-bacterial, and anti-oxidant properties 27, 28. CSR contains many components such as (+)-α viniferin, caraganaphenol A, miyabenol C, and kobophenol A (KPA) 29. KPA is a major active compound of CSR, and it has been studied with respect to osteoblast proliferation 30 and anti-inflammatory 25 activity. CSR appears to inhibit the pathogenesis of OA. Therefore, we aimed to evaluate the protective effect of CSR on interleukin (IL)-1β-stimulated rat chondrocytes and a monosodium iodoacetate (MIA)-induced rat model of OA.

## Materials and Methods

### Reagents

*Caragana Sinica Root* was purchased from “Daehan Yaggug” (Daejeon, Korea). MIA, Protease inhibitor cocktail and phosphatase inhibitor cocktail were purchased from Sigma-Aldrich (St. Louis, MO, USA), Recombinant Rat IL-1β/IL-1F2 Protein and PGE_2_ ELISA kit were purchased from R&D system (Minneapolis, MN, USA). Type II collagen, Trypsin-EDTA (0.25%), Pierce BCA Protein Assay Kit, RIPA lysis buffer and NE-PER™ Nuclear and Cytoplasmic Extraction Reagents were purchased from Thermo (Boston, PA, USA), Primer and AccuPower CycleScript RT PreMix were purchased from Bioneer (Daejeon, Korea), MTT (EZ-Cytox) was purchased from Daeil Lab (Chung-cheong bukdo, Korea), Dulbecco's Modified Eagle's Medium, Fetal bovine serum and Penicillin-Streptomycin (Gibco BRL, USA), 30% Acrylamide/Bis Solution 29:1(3.3%C) and TEMED were purchased from BIO-RAD (Sacramento, CA, USA), Skim Milk was purchased from BD (Denver, Co, USA), Total RNA prep kit, ECL solution and NO assay kit were purchased from Intronbio (Gyeonggi-do, Korea), Primary Antibody ERK, JNK, p38, IκBα, NF-κB and Actin were purchased from cell signaling (Danvers, MA, USA), Primary Antibody SOX-9 was purchased from abcam (London, CAE, England) Peroxidase IgG was purchased from immunoresearch jackson (West Grove, PA, USA), iNOS, COX-2, aggrecan and GAGs were purchased from MyBioSource (San Diego, CA, USA), MMP-3, 9 and 13 were purchased from Elabscience (Houston, TX, USA).

### Preparation of *Caragana Sinica Root*

CSR (50 g) was added to 500 mL distilled water and extracted for 3 h at 100 °C. After extraction, the filtrate was concentrated under reduced pressure with a rotary vacuum evaporator. The concentrated solution was lyophilized with a freeze dryer to obtain 20 g powder. The powder was stored in a deep freezer (-80 °C) and diluted with distilled water at the concentration required for the experiment.

### Quality Control of CSR by High-Performance Liquid Chromatography (HPLC) Analysis

The HPLC analysis was carried out on the Agilent 1260 Infinity II System with Agilent 1260 Infinity Variable Wavelength Detector (VWD). The Agilent eclipse Plus C18 column (250×4.6 mm, 5 µm) was used. The mobile phase consisted of solvent A (0.1% phosphoric acid in H_2_O) and solvent B (0.1% phosphoric in acetonitrile at a flow rate of 1 mL/min). The injection volume of the extract was 10 μL. The elution phase was as follows: 0-35.01 min of 0-80% solvent A and 20-100% solvent B. The elution was monitored at 280 nm.

### Isolation and Culture of Rat Primary Chondrocytes

Chondrocytes were isolated per as previously described 14. Six-week-old, male, Sprague-Dawley rats (170-200 g) were purchased from RaonBio Inc (Gyeonggi-do, Korea). They were housed in plastic cages under controlled temperature and humidity conditions (22 ± 2°C and 55 ± 15 %, respectively) and light-dark cycle (12:12 h). Fresh-air was ventilated 10-15 times per hour, and the animals had *ad libitum* access to food pellets and water. Chondrocytes were isolated from the articular cartilage after excessive respiratory anesthesia in normal rats. The cartilage tissue was cut into small pieces measuring 1×1×1 mm and washed with phosphate buffered saline (PBS). Joint cartilage pieces were treated with 0.25 % trypsin-EDTA solution for 30 min to remove impurities. Next, they were digested in 0.25 % type II collagenase in 8 h at 37 °C. The digested cartilage was collected by centrifugation for 5 min at 1200 rpm at 4 °C. The enriched chondrocytes were in cultured in Dulbecco's Modified Eagle Medium (DMEM) with 10 % fetal bovine serum (FBS) and 1 % penicillin-streptomycin-neomycin in a 5 % CO_2_ incubator at 37 °C. Cell culture was performed once every 2-3 days at 80-90 % density; 0.25 % trypsin EDTA solution was used.

### Cell Viability Assay

Cytotoxicity of CSR was evaluated by MTT assay. Chondrocytes were seeded in 96-well plates with 5×10^3^ cells/well for 24 h. After 24 h, chondrocytes were treated with 100, 300 and 500 μg/mL CSR for 24 h. Then, 10 μL MTT solution was added to each well and incubated for 1 h in the 5 % CO_2_ incubator at 37 °C. The optical density was measured at a wavelength of 450 nm using a micro-plate reader (Molecular Devices Co., USA).

### Quantitative Real-Time Polymerase Chain Reaction (qRT-PCR)

Chondrocytes were seeded in 6-well plates at a density of 3×10^5^ cells/well for 24 h. After 24 h, cells were pre-treated 100, 300, and 500 μg/mL CSR for 1 h and stimulated with or without IL-1β (10 ng/mL) for another 24 h. Isolation of total RNA was done using the Total RNA Prep Kit (Intronbio, Gyeonggi-do, Korea) according to the manufacturer's instructions. The concentration was determined spectrophotometrically at 260 nm (Thermo Scientific NanoDrop 2000). The RNA quality and purity were determined by the ratio of absorbance at OD260: OD280. cDNA was synthesized using 1 μg of total RNA, and the AccuPower CycleScript RT PreMix was purchased from Bioneer (Daejeon, Korea). Real-time PCR was performed using SYBR Green (Qiagen, Hilden, Germany), per the manufacturer's protocols. The PCR conditions were as follows: Initial denaturation at 95 ˚C for 5 s, primer annealing followed by 40 cycles at 62.5 ˚C for 30 s. mRNA levels were normalized through the housekeeping gene actin. Expression of each target gene was calculated using the 2-ΔΔCT method. The primer sequences are listed in **Table 1.**

### Western Blotting

Chondrocytes were seeded in 6-well plates at a density of 3×10^5^ cells/well for 24 h. After 24 h, cells were pre-treated with 100, 300, and 500 μg/mL CSR for 1 h and stimulated with or without IL-1β (10 ng/mL) for another 24 h. Total cell proteins were extracted using RIPA lysis buffer, reaction on ice, and centrifuged for 30 min at 12,000 rpm at 4 °C. Nuclear and cytosol proteins were lysed using the NE-PER nuclear and cytoplasmic extraction kit, per the manufacturer's instructions. Then, proteins were measured using the bicinchoninic acid (BCA) protein assay kit. Proteins (30 µg/well) were separated on 10 % sodium dodecyl sulfate-polyacrylamide gel (SDS-PAGE) and transferred to polyvinylidene fluoride (PVDF) membranes. The membranes were incubated overnight at 4 °C in 5% skim milk blocking buffer and primary antibodies against ERK1/2, phosphorylation-ERK1/2 (1:1000), JNK, phosphorylation-JNK (1:1000), P38, phosphorylation-P38 (1:1000), IκBα (1:500), NF-κB p65 (1:500), SOX-9 (1:500), and Actin (1:1000) Next, the membranes were washed with TBST and incubated with secondary antibodies (1:10000) at room temperature for 1 h. Enhanced chemiluminescence (ECL) kit was used for detection and the membranes were visualized using a ChemiDoc (Vilber Lourmat, France).

### Animal Experiments

Rats were housed as detailed in section 2.4. All experimental procedures complied with the National Institute of Health Guide for the Care and Use of Laboratory Animals and the Korean National Animal Welfare Law. The experimental animal facility and protocols were approved by Daejeon University Institutional Animal Care and Use Committee (DJUARB2019-003). The rats were rested for one week to adjust to the environment before the experiment. The rats were randomly divided into five groups of six rats each. For surgery, monosodium iodoacetate (MIA) solution (3 mg/100 μL in 0.9 % saline) was injected using an insulin syringe into the intra-articular space of the right knees of rats. The rats were treated as indicated: Normal (DW), control (MIA 3 mg/100 μl and DW), positive control (MIA 3 mg/100 μl and indomethacin 1 mg/kg), CSR 200 (MIA 3 mg/100 μl and CSR 200 mg/kg), and CSR 400 (MIA 3 mg/100 μl and CSR 400 mg/kg). CSR was orally administered for 4 weeks after MIA injection. All rats were sacrificed on day 29 post treatment.

### Measurement of Serum Nitric Oxide and PGE_2_ Production

On the day of sacrifice, whole blood samples were collected and the blood was allowed to coagulate for 30 min. Then, the serum was separated by centrifugation for 10 min at 2,000 rpm at 4 °C and stored at ‑80 ˚C until further use. Productions of NO in the serum samples were determined using the Griess reaction for nitric oxides. Briefly, 100 µl serum was mixed with 50 µl Griess reagent A and 50 µl Griess reagent B, followed by incubation for 15 min at 37 ˚C. Optical density was measured at 540 nm on an ELISA reader. Productions of PGE2 in the serum samples were determined using an ELISA kit (R&D Systems), per the manufacturer's instructions.

### Measurement of Serum Bio-Markers

The levels of MMP-3, 9, and 13 were measured using assay kits from Elabscience. Expression of iNOS, COX-2, aggrecan and GAGs were determined using assay kits from MyBioSource. All assays and procedures were performed per the manufacturers' instructions.

### Histological Analysis and Score

The knee joint was collected on the day of sacrifice. The samples were fixed in 10 % formalin for 1 day, decalcified using 10 % EDTA for 2 weeks, dehydrated in a graded series of ethanol, washed, and embedded in paraffin blocks. Then, 5-μm-thick sections were cut from the embedded paraffin block and stained with Harris hematoxylin & eosin and Safranin-O/Fast Green. Images were captured using a light microscope (Axioskop 40; Carl Zeiss AG, Germany) and photographed (Axiocam MRc5; Carl Zeiss AG, Germany). Cartilage degeneration change was scored using the Osteoarthritis Research Society International (OARSI) guidelines.

### Micro-CT Analysis of Knee Cartilage

After the experiment, the right femur and tibia were cut and fixed in 10% formalin by removing the skin and muscles. Then, the tissues were commissioned for micro-CT analysis at KPNT (Cheongju, Korea), an inspection institution. The cartilage region was evaluated by converting the micro-CT imaging results into 3D images.

### Statistical Analysis

Data are presented as the mean ± SD. Statistical analysis was performed using Graph Pad PRISM Software (Graphpad Software Inc, CA, USA). One-way ANOVA was used to evaluate the treatment effect, followed by Student's t-test. Values of *p <* 0.05 were considered significant.

## Results

### HPLC Analysis

**Figure 1:**
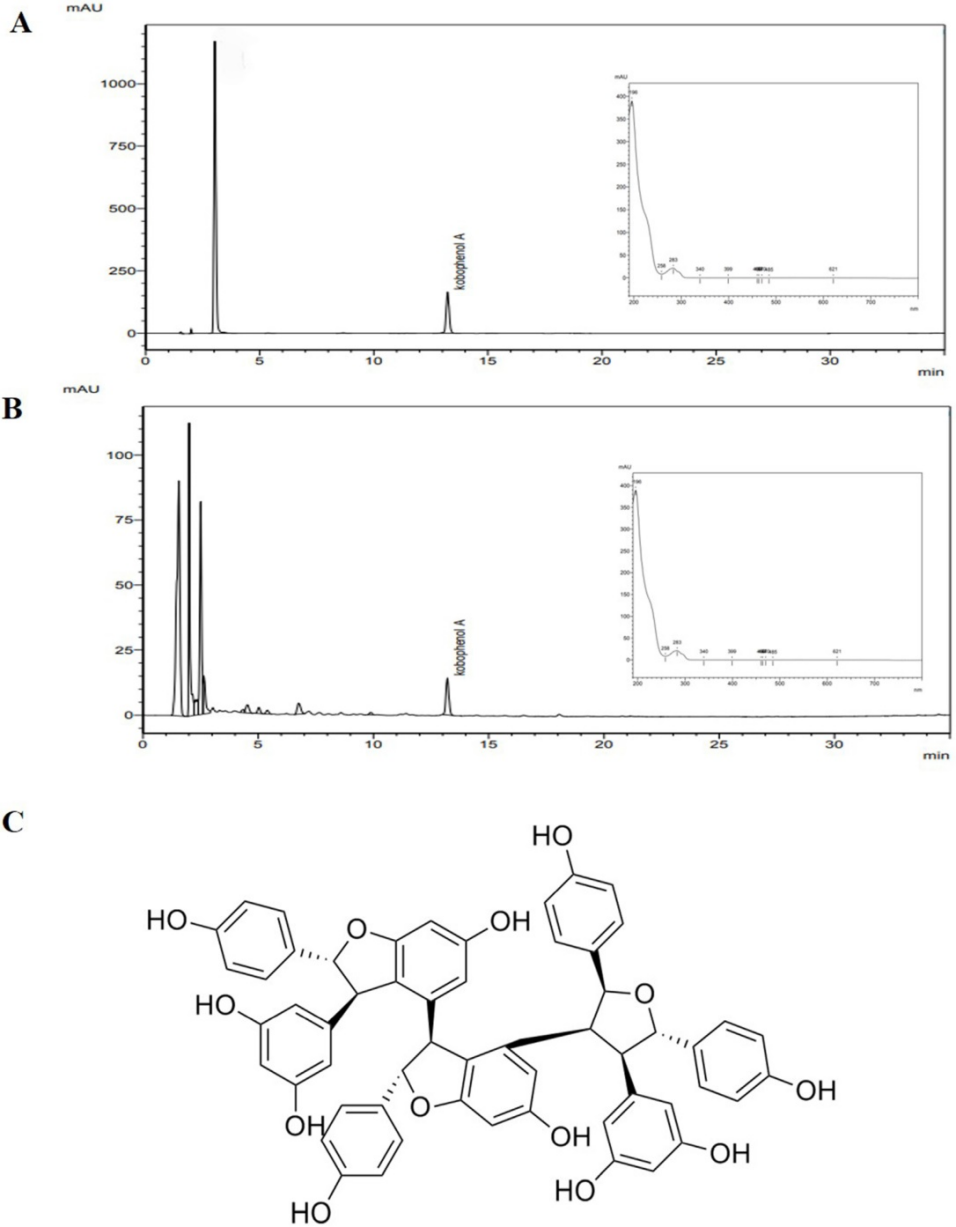
** Representative HPLC chromatograms of (A)** Kobophenol A **(B)** CSR detected at 280 nm and the **(C)** chemical structures of its major compounds.

Kobophenol A (KPA) was used as a standard compound of CSR. As shown Fig. 1, the retention time of the KPA was 13.232 min and contained 200.487 mg/g (Fig. 1A). The chromatographic peak of the CSR was 13.205 min in a wave-length of 280 nm (Fig. 1B). Fig. 1C shows the chemical structure of Kobophenol A (KPA).

### Inhibitory Effects of CSR on MMPs and ADAMTSs mRNA Expression in IL-1β-Stimulated Chondrocytes

**Figure 2:**
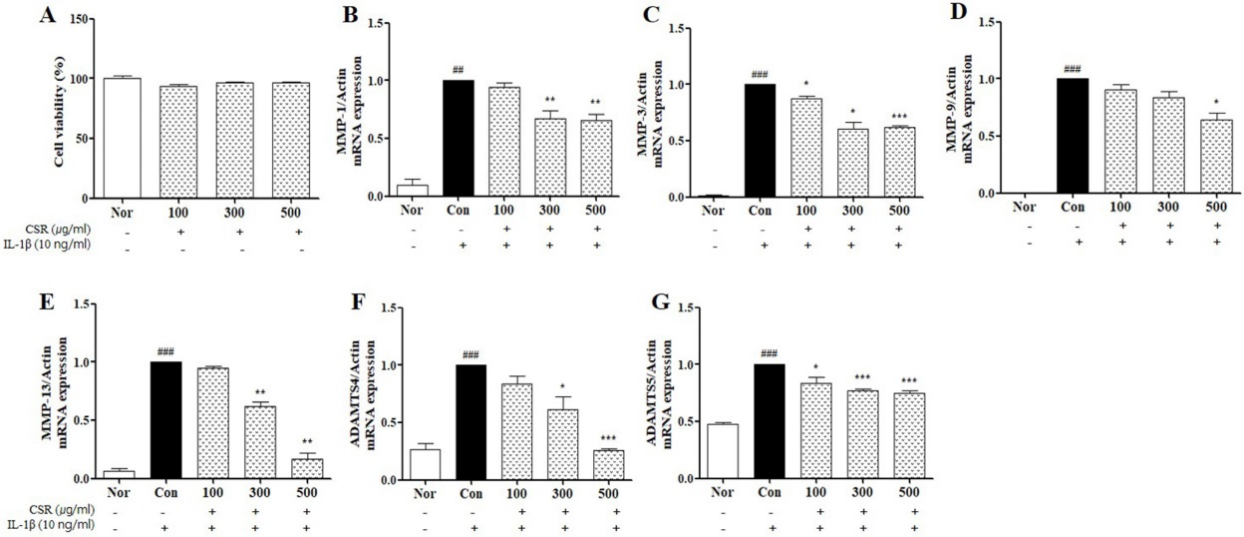
** The effects of CSR on MMPs, ADAMTS mRNA expressions in IL-1β-stimulated chondrocytes. (A)** Cytotoxicity of CSR was determined by MTT assay. The level of mRNA expression of **(B)** MMP-1, **(C)** MMP-3, **(D)** MMP-9, **(E)** MMP-13, **(F)** ADAMTS4 and **(G)** ADAMTS5 was examined by real-time PCR. ^###^*p <* 0.001, ^##^*p <* 0.01 compared with normal and ^***^p < 0.001, ^**^p < 0.01, ^*^p < 0.05 compared with the control group.

Cytotoxicity of CSR on chondrocytes was evaluated using an MTT assay. None of the tested concentrations of CSR (100, 300, and 500 μg/mL) were cytotoxic to chondrocytes (Fig. 2 A). Therefore, we chose a concentration range of 100-500 μg/mL for subsequent experiments.

To investigate the expression of catabolic enzymes in response to CSR treatment in IL-1β-stimulated chondrocytes, we examined the effect of CSR on MMP-1, MMP-3, MMP-9, MMP-13, ADAMTS4 and ADAMTS5 by real-time PCR. Enzyme concentrations of catabolic factors (MMP-1, MMP-3, MMP-9, MMP-13, ADAMTS4 and ADAMTS5) were up-regulated as compared to the normal group. However, a dose-dependent decrease in enzyme levels was noted upon CSR treatment compared to the control group (Fig. 2 B-G).

### Effects of CSR on ECM mRNA Expression in IL-1β-Stimulated Chondrocytes

**Figure 3:**
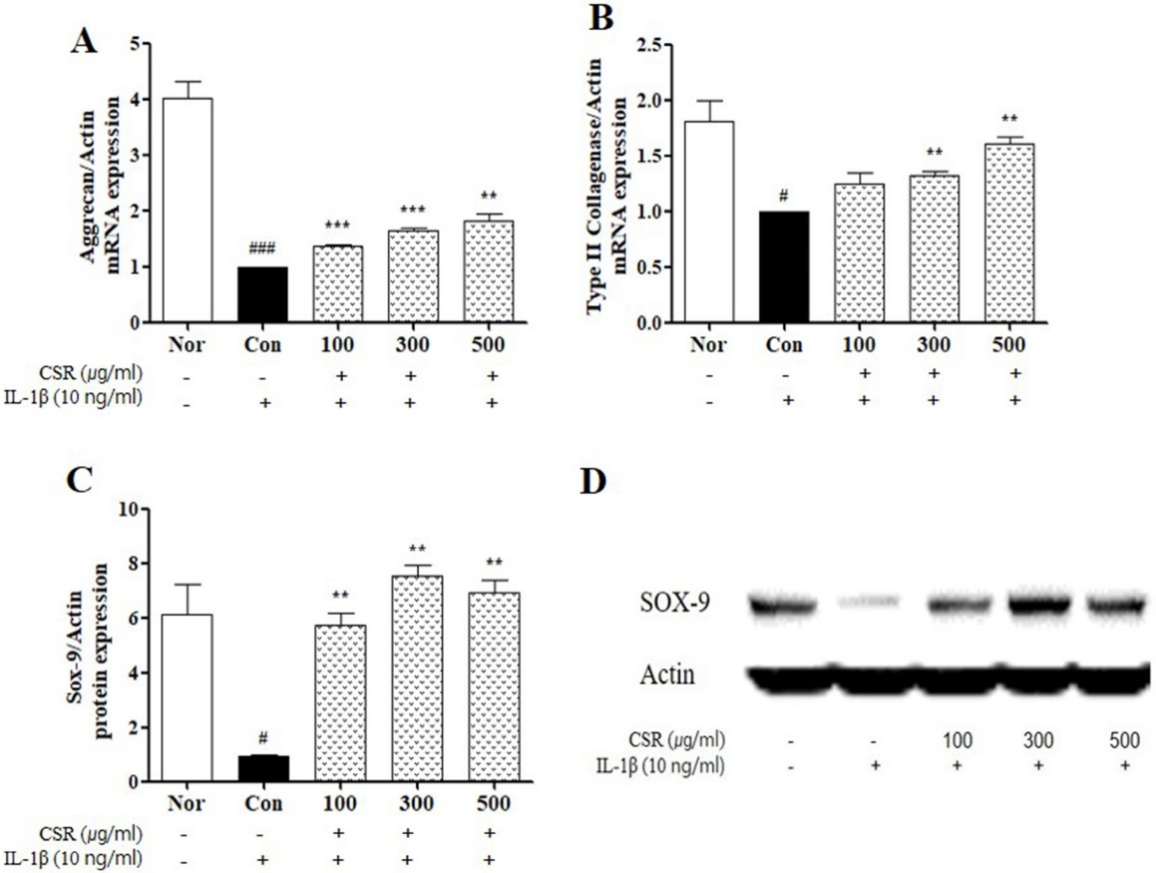
** The effects of CSR on ECM mRNA expressions SOX-9 protein expressions in IL-1β-stimulated chondrocytes. (A)** Aggrecan and **(B)** Type Ⅱ Collagenase was examined by real-time PCR. **(C)** SOX-9 was examined by Western blot. **(D)** Evaluation of protein expression by image J. Each dataset represents the mean±SEM. ^###^*p <* 0.001, ^#^*p <* 0.05 compared with normal and ^***^p < 0.001, ^**^p < 0.01 compared with the control group.

To evaluate the effect of CSR on cartilage degradation in IL-1β-stimulated chondrocytes, we detected the expression of aggrecan and type II collagen by real-time PCR and of SOX-9 by western blotting (Fig. 3 A-C). The expression of aggrecan, type II collagen, and SOX-9 was down-regulated upon CSR treatment as compared to the normal group. However, compared to the control group, CSR treatment increased the expression of aggrecan, type II collagen, and SOX-9 compared to the control group (Fig. 3 D).

### Effects of CSR on the Inhibition of MAPK/NF-κB Protein Expression in IL-1β-Stimulated Chondrocytes

**Figure 4:**
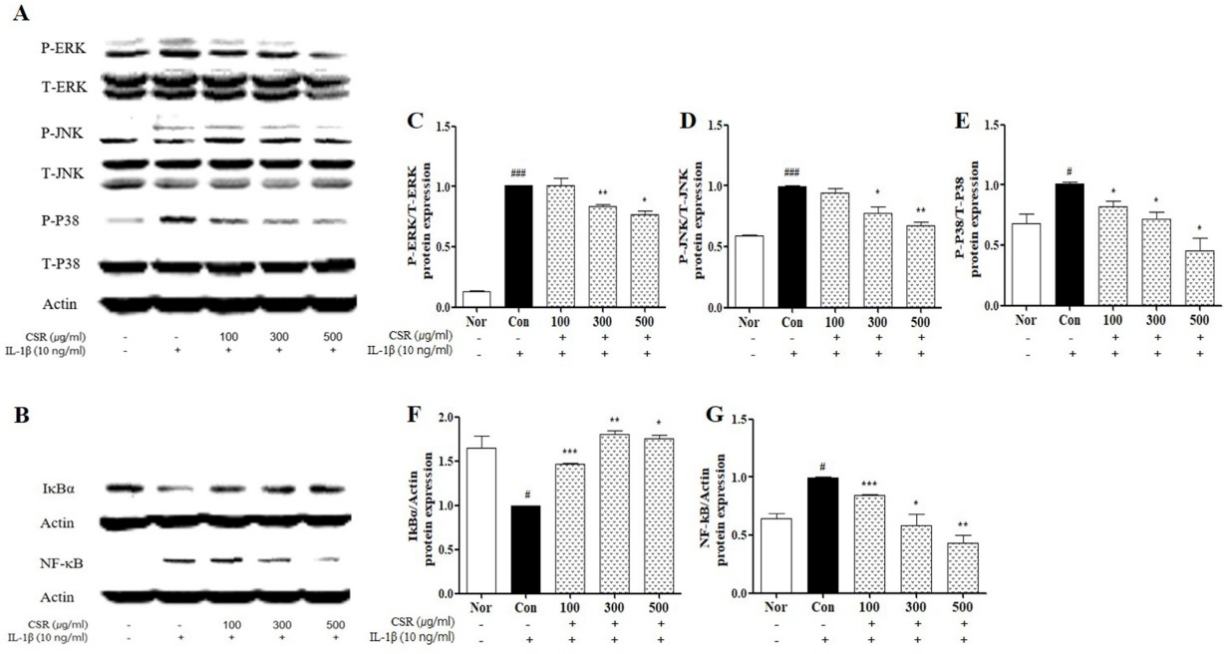
** The effects of CSR on MAPK/ NF-κB signaling protein expressions in IL-1β-stimulated chondrocytes. (A, B)** The level of NF-κB, phosphor-ERK1/2, JNK, P38 was examined by Western blot. **(C-G)** Evaluation of protein expression by image J. Each dataset represents the mean±SEM. ^###^*p <* 0.001, ^#^*p <* 0.05 compared with normal and ^***^p < 0.001, ^**^p < 0.01, ^*^p < 0.05 compared with the control group.

To evaluate the effect of CSR on MAPK/NF-κB signaling pathway in IL-1β-stimulated chondrocytes, we detected the phosphorylation levels of ERK, JNK, p38 and NF-κB/IκBα by western blotting (Fig. 4 A,B). MAPK, an NF-κB regulator, was up-regulated compared to normal group. The CSR-treated groups showed a dose-dependent decrease in the MAPK expression, as compared to the control group (Fig. 4 c-e). Furthermore, NF-κB and IκBα were inhibited concentration-dependently of activity of NF-κB and IκBα degradation in group treated with CSR (Fig. 4 F, G).

### Effects of CSR on Serum Inflammatory Mediators and MMPs levels in a Rat Model with MIA-Induced OA

**Figure 5:**
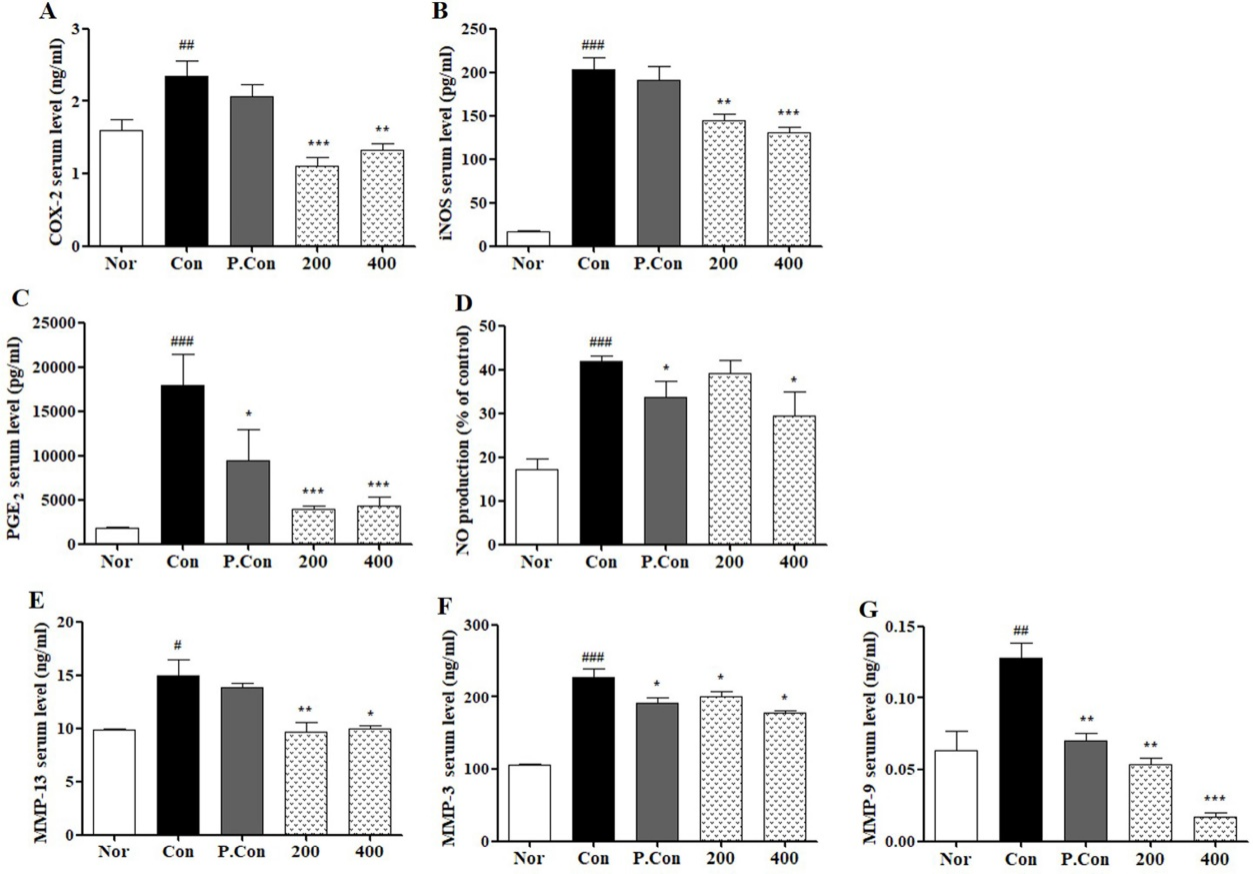
** The effects of SCR on serum COX-2, iNOS, PGE_2_, NO, MMP-13, MMP-9 and MMP-3 level in MIA-induced SD-Rat.** The levels measure of total serum **(A)** COX-2, **(B)** iNOS, **(C)** PGE_2_, **(D)** NO, **(E)** MMP-13, **(F)** MMP-3 and **(G)** MMP-9 were examined by ELISA kit and NO assay kit. Each data represents the mean SEM. ^###^*p <* 0.001, ^##^*p <* 0.01, ^#^*p <* 0.05 compared with normal and ^***^p < 0.001, ^**^p < 0.01, ^*^p < 0.05 compared with control.

To assess the effect of CSR on inflammatory mediators in MIA-induced OA, we detected serum levels of COX-2, PGE_2_, iNOS, and NO by ELISA and NO Assay (Fig. 5 A-D). Serum levels of COX-2, PGE_2_, iNOS, and NO were greater in the control group than the normal group. However, the CSR and P.Con treated groups showed lower serum levels than the control group.

To investigate the effect of CSR on catabolic enzymes, we determined the serum levels of MMP-3, MMP-9 and MMP-13 by ELISA (Fig. 5 E-G). Enzymes of catabolic factors (MMP-3, MMP-9, MMP-13) were up-regulated in control group compared to normal group, while those factors were decreased in CSR and P.Con groups.

### Effects of CSR on Serum Aggrecan and GAGs levels in a Rat Model of MIA-Induced OA

**Figure 6:**
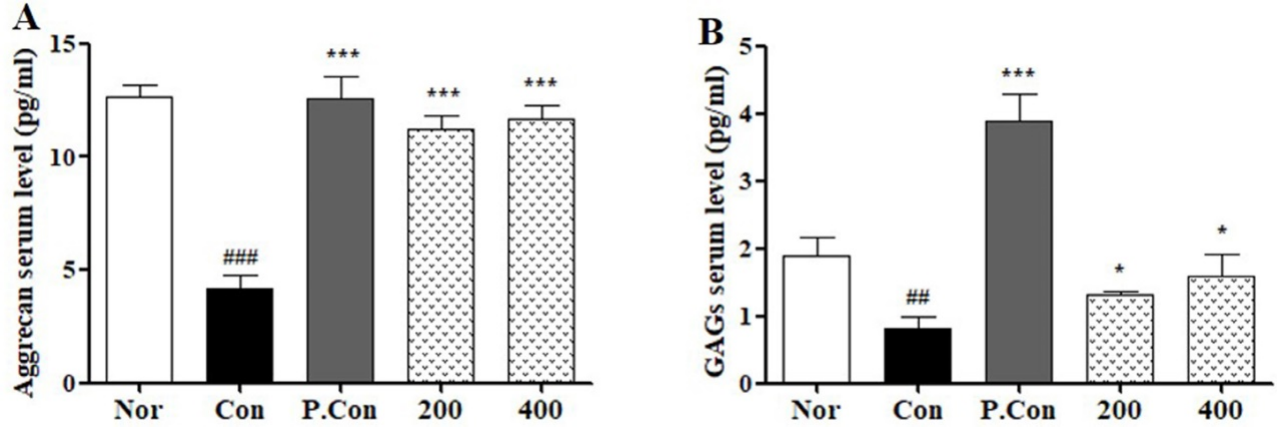
** The effects of SCR on serum ECM level in MIA-induced SD-Rat. The levels measure of total serum (A)** Aggrecan and **(B)** GAGs were examined by ELISA kit. Each data represents the mean SEM. ^###^*p <* 0.001, ^##^*p <* 0.01 compared with normal and ^***^p < 0.001, ^*^p < 0.05 compared with control.

To evaluate the effects of CSR on cartilage matrix degradation in MIA-induced OA, we detected serum levels of aggrecan and GAGs by ELISA (Fig. 6 A,B). The levels of aggrecan and GAGs were decreased in the control groups compared to the normal group. In contrast, the CSR and P.Con treated groups showed increase in serum levels of aggrecan and GAGs as compared to the control group.

### Effects of CSR on Knee Joints Destruction in a Rat Model of MIA-Induced OA

**Figure 7:**
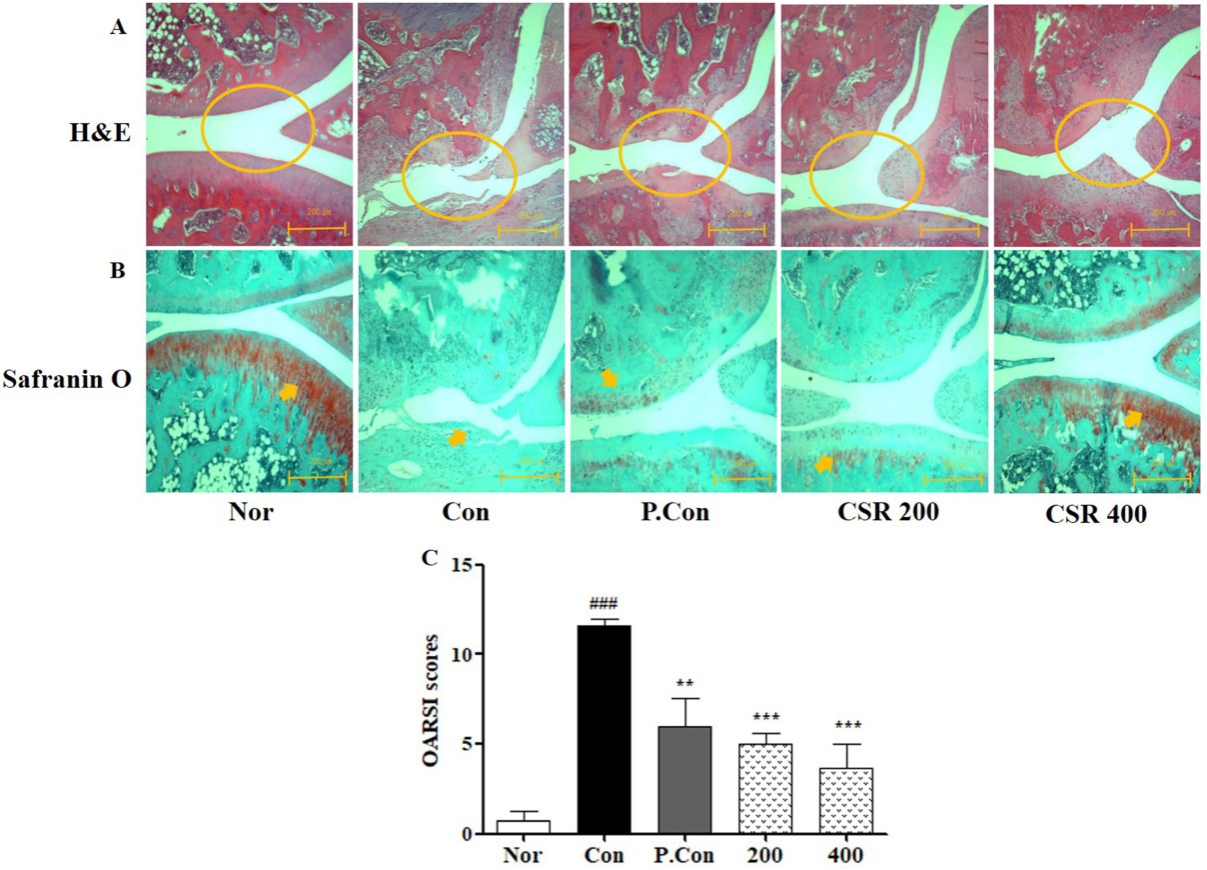
** The effects of CSR on knee joints' destruction in MIA-induced OA. (A)** Cartilage preservation, **(B)** proteoglycan distribution, and **(C)** OARSI scores as examined by H&E and Safranin O staining. Each dataset represents the mean±SD. ^###^*p<*0.001 compared with the normal group and ^***^p<0.001, ^**^p<0.01 compared with the control group.

To evaluate the protective effect of CSR on knee cartilage in MIA-induced OA, we performed histochemical analysis of destruction of cartilage, thinner cartilage layers, and rough surfaces by H&E staining (Fig. 7 A) and proteoglycan around the cartilage by Safranin O staining (Fig. 7 B). CSR and P.Con inhibited the observed cartilage degradation, as well as slowed OA progression. Consistent with these findings, the OARSI score in the CSR and P.Con treated groups was lower than that of the control group (Fig. 7 C).

### Effects of CSR on the Femur and Articular Cartilage Volume in a Rat Model of MIA-Induced OA

**Figure 8:**
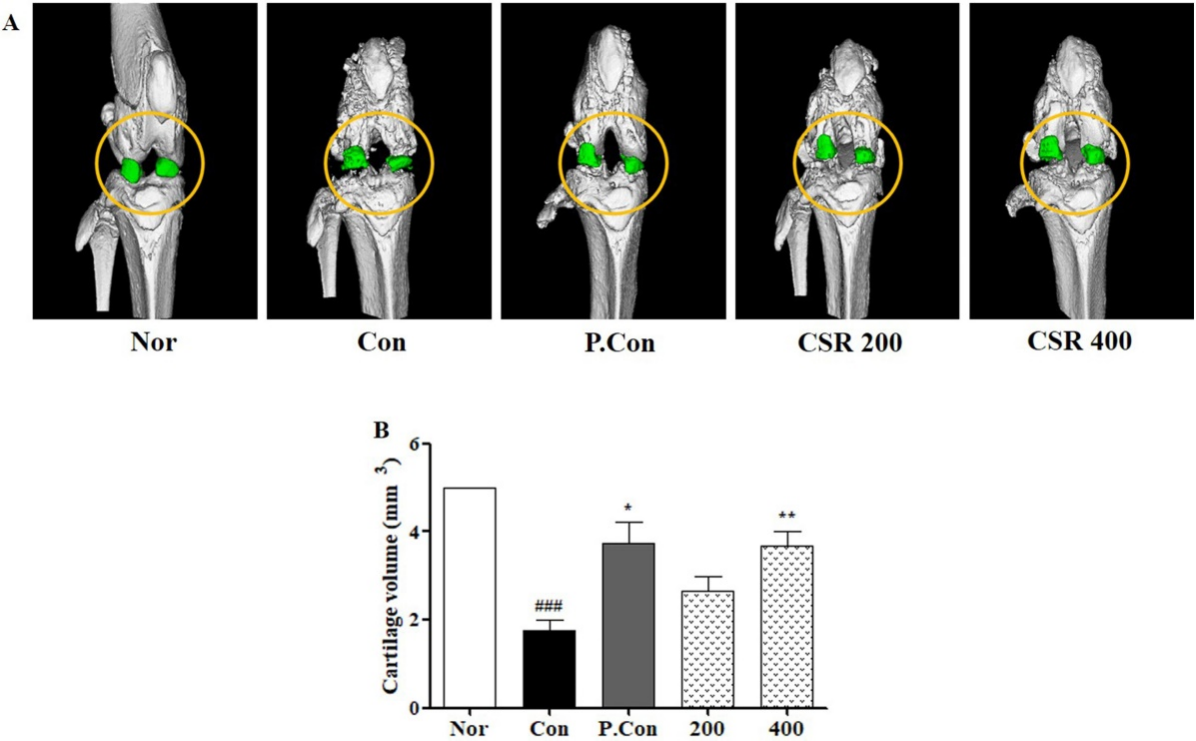
** The effects of CSR on the femur and knee joints' volume in MIA-induced OA. (A)** Three-dimension micro-CT images and **(B)** cartilage volume were examined by the micro-CT system. Each dataset represents the mean±SD. ^###^*p<*0.001 compared with the normal group and ^**^p<0.01, ^*^p<0.05 compared with the control group.

To understand the microarchitecture of knee joints after CSR treatment in MIA-induced OA, we carried out morphological analysis of the femur and articular cartilage volume by micro CT (Fig. 8 A). CSR and P.Con treatment inhibited the observed femur bone architecture as well as showed less cartilage volume compared to the control group (Fig. 8 B).

## Discussion

**Figure 9:**
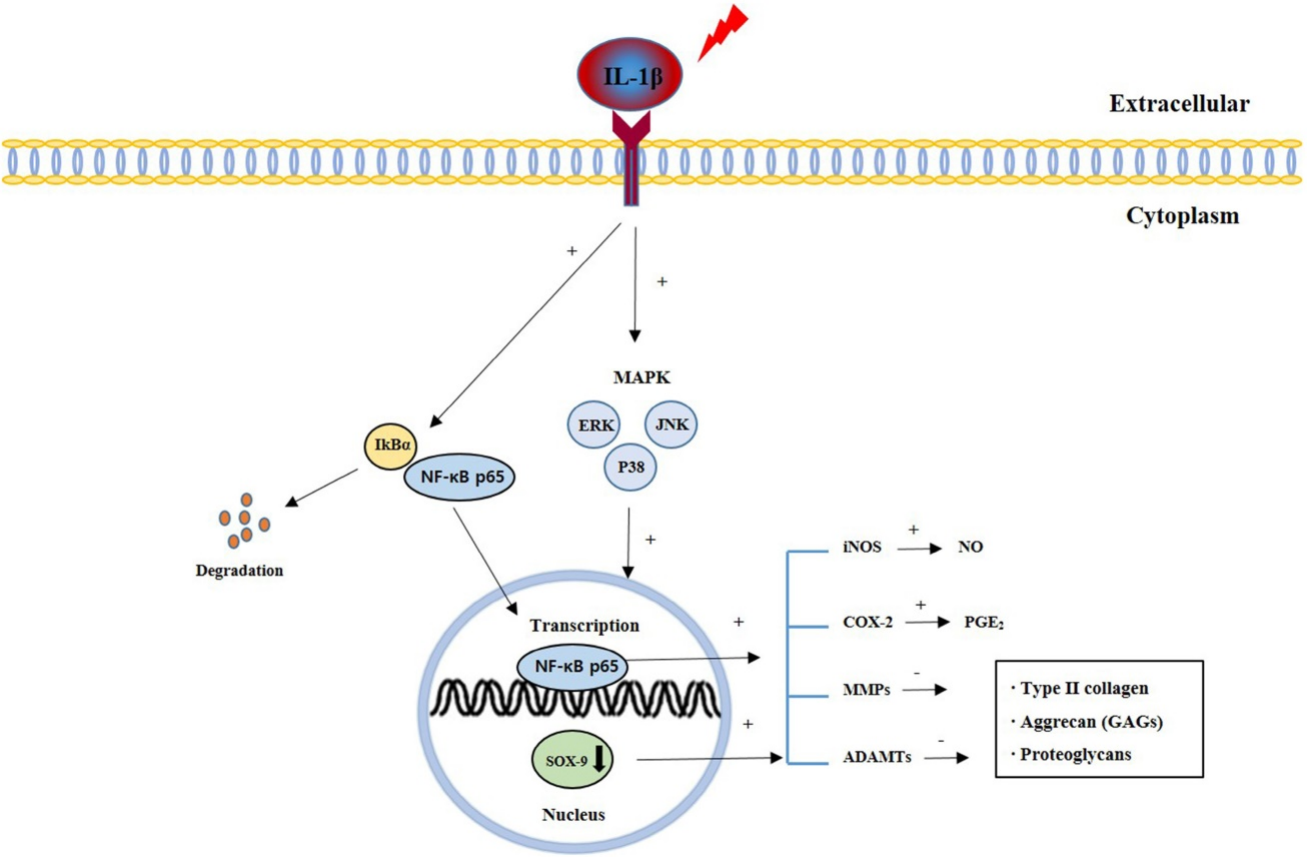
Signal pathways of cartilage degradation during OA pathogenesis and progression.

The present study investigated the effects of the CSR extract on the progression of OA, both *in vitro* and *in vivo*. IL-1β is a pro-inflammatory cytokine involved in OA and is involved in cartilage degradation 8. The CSR extract improved the synthesis and reduced degradation of ECM by blocking the MAPK/NF-κB signaling pathway in IL-1β-stimulated chondrocytes isolated from rat knee articular cartilage. MIA is a promoter that causes necrosis of chondrocytes and causes arthritis similar to degenerative arthritis. The CSR extract improved the synthesis and reduced degradation of ECM and was had an effect on inflammatory mediator, histopathological analyses, and micro-CT analysis in a rat model of MIA-induced OA (Fig. 9).

NO is well-known inflammatory mediator and is produced by the nitric oxide synthase (NOS) family of enzymes 31. PGE_2_ is a major mediator of pain and inflammation in OA and is elevated by COX-2 32, 33. NO and PGE_2_ play important roles in the pathogenesis of OA, such as stimulating the synthesis and activity of MMPs and inhibiting the production of ECM in cartilage. Inhibition of NO and PGE_2_ production may be a useful treatment option to delay the progression of OA 34, 35. Moreover, previous studies have reported that inhibiting the production of COX-2 and iNOS may protect the articular cartilage 36, 37. In the present study, the levels of iNOS, COX-2, NO, and PGE_2_ were significantly increased in the serum of the rat model of MIA-induced OA. CSR treatment led to decreased serum levels of iNOS, COX-2, NO, and PGE_2_. These results suggested that the anti-inflammatory effect of CSR could delay cartilage degradation owing to the inhibitory effect of inflammatory mediators.

MMPs and ADAMTSs are catabolic enzymes 38. MMPs are key proteolytic enzymes that cause cartilage destruction in OA and are known to regulate the destruction of cartilage matrix components such as type II collagen, aggrecan, and various proteoglycans 39, 40. In OA, family of MMPs such as MMP-1, MMP-3, MMP-9, and MMP-13 increase 8. MMP-1 and especially MMP-13 are known to play a major role in the degradation of type II collagen, aggrecan, and proteoglycans in OA cartilage 41. In addition, MMP-3 can help MMP-1 and MMP-13 to degrade cartilage components; MMP-9 is similar to MMP-1 and is essential for chondrocyte apoptosis 8, 41. Cartilage components—type II collagen and aggrecan are essential for maintaining normal chondrocyte metabolism 42. When cartilage degeneration occurs, the chondrocytes may also undergo apoptosis 43, 44. Apoptotic cells of chondrocytes reduce the production of type II collagen and aggrecan and increase proteolytic enzymes, leading to cartilage destruction 39, 43. Furthermore, aggrecan induces the release of GAGs and, which bind to aggrecan core proteins to form proteoglycans and play a role in cartilage elasticity 45. Besides MMPs, cleavage of aggrecan can be mediated by several members of ADAMTS such as ADAMTS-4 and ADAMTS-5 in OA pathogenesis 46. ADAMTS members play an important role in the degradation of proteoglycans and loss of GAGs 47. Both ADAMTS-4 and ADAMTS-5 are key enzymes for cleaving aggrecans, but only ADAMTS-5 is active 48, 49. In our study, the effects of CSR on the mRNA levels of MMP-1, MMP-3, MMP-9, MMP-13, ADAMTS-4, ADAMTS-5, aggrecan, and type II collagen were evaluated in IL-1β-stimulated chondrocytes. CSR treatment inhibited MMP-1, MMP-3, MMP-9, MMP-13, ADAMTS-4, and ADAMTS-5 expression and degradation of aggrecan and type II collagen. Furthermore, the levels of MMP-3, MMP-9, MMP-13, aggrecan, and GAGs were confirmed in the serum of MIA-induced OA rats. CSR inhibited the MMP-3, MMP-9, MMP-13, degradation of aggrecan and GAGs. These results suggest that CSR has chondroprotective effects by suppressing the synthesis of catabolic enzymes.

The *SOX-9* gene is one of the most important transcription factors for cartilage formation and maintenance of chondrocytic phenotype 50, 51. SOX-9 regulates the expression of cartilage matrix genes such as aggrecan and type II collagen 52, 53. In addition, up-regulation of SOX-9 mediated inhibition of ADAMTSs 54. In the present study, the expression of SOX-9 was significantly decreased in IL-1β-stimulated chondrocytes. CSR increased the protein expression of SOX-9. These results suggest that CSR can play an important role in the regulation of chondrocyte development given the improvements in SOX-9 as well as ADAMTSs, aggrecan, and type II collagen.

MAPKs (phosphorylation of ERK, JNK, and p38) and NF-κB are important signaling pathways in OA pathogenesis 14. MAPKs activity is related in the process regulation of cartilage degradation and MMP expression and is involved in cellular activities such as cell subdivision, proliferation, and survival 55, 56. The transcription factor NF-κB pathway is activated by phosphorylation of MAPKs and is involved in the regulation of inflammatory mediators as well as OA progression 57. In the inactive state, NF-κB is localized to the cytoplasm with its inhibitor subunit IκBα 58. After stimulation by IL-1β, NF-κB is separated from the IκBα subunit and is translocated to the nucleus 59. After nuclear translocation, NF-κB upregulates inflammatory mediators and catabolic enzymes that contribute to OA progression 49, 59. In this study, the protein expression of MAPKs, degradation of IκBα, and translocation of p65 were significantly promoted in IL-1β-stimulated chondrocytes. These results suggest that the MAPKs/NF-κB signaling pathway is affected by CSR treatment and could be the primary molecular pathway in OA pathology and can be an effective target for therapy.

Furthermore, CSR brought about tissue-specific changes as seen by histological and micro-CT analysis to objectively assess the physiological and pathological status of the affected cartilage. H&E staining identified destruction of cartilage, thinner cartilage layers, and rough surfaces. Safranin O staining confirmed proteoglycan around the cartilage. Histological analysis showed that control animals with MIA-induced OA showed more abnormalities in proteoglycan distribution, cartilage thickness, synovial membranes, and fibrous tissue than the normal animals; whereas, the CSR-treated group suppressed cartilage deformity, increased proteoglycans, and normalized the synovial membrane and fibrous tissue as compared to the control group. This result was consistent with the OARSI score. Finally, micro-CT analysis of the cartilage volume of the knee joint showed that the volume of cartilage was greater in the CSR-treated groups than the control group. Furthermore, the components of CSR are needed a mechanism study for OA inflammatory processes.

## Conclusion

Our results demonstrated that CSR reduced ECM degradation and catabolic enzymes by inhibition of NF-κB and MAPKs signaling pathways in IL-1β-stimulated chondrocytes. Also, CSR reduced the inflammatory mediators, catabolic enzymes and ECM degradation in the rats with MIA induced OA. Furthermore, CSR treatment also decreased the OARSI scores in addition to the extent of synovitis. Overall, CSR can be a potent drug for the treatment of OA via the decreased catabolic enzyme synthesis and increased anabolic enzyme secretion.

## Figures and Tables

**Table 1:** Primer sets for RT-PCR

Name	Forward	Reverse
MMP-1	5′ AGGTGTGGGGTGCCTGATGT 3'	5' TCCTCCTCAA AAACCCTTTC 3'
MMP-3	5' TTTGATGTACCCAGTCTACA3'	5' TGACTGCATC GAAGGACAAA 3'
MMP-9	5' CCAGGAGTCTGGATAAGTTG 3'	5' ACGCTCTGGGGATCCACCTT3'
MMP-13	5' TGACACCTCTGAATTTTACC 3'	5' CCGCCAAGGTTTGGTCCAGG 3'
ADAMTS4	5' GCCAGCAACC GAGGTCCCAT 3'	5' TTGGCAGCGG CGGCCATGAC 3'
ADAMTS5	5' CCGCACCTCG AAACAGTGGC 3'	5' CACCTGCGTA TTTGGGAACC 3'
Type II collagen	5'GAGTGGAAGAGCGGAGACTACTG 3'	5'GTCTCCATGTTGCAGAAGACTTTCA 3'
Aggrecan	5' CAGAAACCTA TGATGTCTAC 3'	5' CAGCCAGCAT AGCACTTGTC 3'
Actin	5' GACGGTCAGG TCATCACTAT 3'	5' GGTACATGGT GGTGCCACCA 3'

## Abbreviations

ADAMTS: A Disintegrin and Metalloproteinase with Thrombospondin motifs
CSR: *Caragana sinica root*
COX-2: Cyclooxygenase-2
ECM: Extracellular matrix
GAGs: Glycosaminoglycans
IL-1β: Interleukin-1β
IκBα: Inhibitor of kappa b alpha
iNOS: Inducible nitric oxide synthase
KPA: kobophenol A
NF-κB: Nuclear factor-κB
NO: Nitric oxide
NSAIDs: Nonsteroidal anti-inflammatory drugs
MIA: Monosodium iodoacetate
MARK: Mitogen-activated protein kinase
MMPs: Matrix metalloproteinases
OARSI: Osteoarthritis Research Society International
PGE_2_: Prostaglandin E2
p-ERK: Phosphorylation-ERK
p-JNK: Phosphorylation-JNK
p-P38: Phosphorylation-P38
OA: Osteoarthritis
